# The levels of the sex hormones are not different between type 1 and type 2 endometrial cancer

**DOI:** 10.1038/srep39744

**Published:** 2016-12-21

**Authors:** Jiayi Wan, Yifei Gao, Ke Zeng, Yongxiang Yin, Min Zhao, Jia Wei, Qi Chen

**Affiliations:** 1 Department of Pathology, Wuxi No 2 People’ Hospital, Nanjing Medical University, China; 2 The Hospital of Obstetrics & Gynaecology, Fudan University, China; 3 Department of Gynae-oncology, Wuxi Maternity and Children Hospital, Nanjing Medical University, China; 4 Department of Obstetrics & Gynaecology, The University of Auckland, New Zealand

## Abstract

The involvement of hormonal factors in developing endometrial cancer is well documented. In particular, excess or unopposed estrogen is a major risk factor. Endometrial cancer is divided into estrogen-dependent and estrogen-independent types. Studies suggested that the subtypes of endometrial cancer share many common risk factors. Whether the levels of sex hormones differ between types 1 and 2 endometrial cancer has not been investigated. In this study, levels of estrogen, progesterone, testosterone, follicle stimulating hormone (FSH) and luteinizing hormone (LH) were investigated between type 1 and type 2 endometrial cancer taking into account menopausal status and parity. The sex hormones levels and estrogen and progesterone receptors were measured in 187 women with endometrial cancer. The levels of estradiol (E2), progesterone, testosterone, FSH and LH were not different between the subtypes of endometrial cancer regardless of menopausal status. In addition, the sex hormones were not different between patients of different party regardless of the menopausal status. The majority of type 1 (96%) and type 2 (82%) endometrial cancers were estrogen and progesterone receptor positive. Our data suggest that type 2 endometrial cancer is not completely estrogen independent, and type 1 and type 2 endometrial cancers may have a similar pathogenesis.

Endometrial cancer is the most prevalent cancer of the female reproductive tract in developed countries. In 2012, 320,000 women were diagnosed with endometrial cancer, resulting in 76,000 deaths worldwide^1^. Endometrial cancer is the fourth most common cancer among women in the United States^2^ and the sixth most common cancer in women worldwide (World Cancer Report 2014, World Health Organization). The exact causes of endometrial cancer are still unclear, however a number of risk factors for developing endometrial cancer such as early menarche and late menopause, nulliparity, obesity, increasing age, hypertension and ethnicity have been identified^3^,^4^,^5^,^6^,^7^,^8^.

A shift in the balance of estrogen and progesterone towards a more hyperestrogenic state increases the risk for developing endometrial cancer^9^. An estimated 40% of endometrial cancer patients are observed to be obese (World Cancer Report 2014, World Health Organization). In obesity, excess adipose tissue increases the conversion of androstenedione into estrogens, and a reduction in the clearance of estrogen from the blood is also seen in obese women^10^,^11^. Higher serum levels of estrogen may lead to infrequent or anovulation. In addition, obesity also causes anovulation which consequently reduces progesterone protection of the endometrium from high levels of unopposed endogenous estrogen. This results in continuous exposure of the endometrium to high levels of estrogen^10^,^11^.

Endometrial cancer is traditionally categorised into type 1, which is an estrogen-dependent condition accounting for 80–85% of total cases, and type 2, which is an estrogen-independent condition based on clinical features and pathogenesis^12^. In addition to the differences in histology, the risk factors and clinical outcomes including survival are different depending on the subtype. Prolonged exposure to high levels of circulating estrogen in combination with decreased progesterone is associated with an increased risk of type 1 endometrial cancer^10^. In contrast, type 2 endometrial cancer, which usually occurs in older and post-menopausal women, may not be associated with exposure to unopposed estrogen^13^. A number of studies have recently suggested that parity, a well-known protective factor for the development of endometrial cancer due to the changes in sex hormones levels and it is implied that the parous women are less likely to have anovulation, polycystic ovarian disease or be markedly obese. However, parity has been found to differ in studies that have investigated its relationship to the subtypes of endometrial cancer^14^,^15^. In addition, other studies have suggested that the subtypes of endometrial cancer share many common risk factors, and it has been hypothesised that type 2 endometrial cancer may not be completely estrogen-independent^16^. This prompted us to question whether the levels of sex hormones are in fact different in the different types of endometrial cancer. Therefore, this study aimed to investigate the levels of most common sex hormones including estrogen, progesterone, testosterone, follicle stimulating hormone (FSH) and luteinizing hormone (LH), between type 1 and type 2 endometrial cancer taking into account menopausal status.

## Results

### Clinical characteristics of the study population

The median age of patients at diagnosis was 56 (range 24–82) years old. Of 187 patients, 163 (87%) were diagnosed with type 1 endometrial cancer, and the remaining 13% were diagnosed with type 2 endometrial cancer. Sixty seven (35.8%) patients were diagnosed before menopause. Of the 187 patients, 8 (4.3%) were nulliparous and 105 (56.1%) patients had one live birth. There was no statistical difference in the median age between premenopausal women with type 1 (47 range from 24 to 67 years) and type 2 endometrial cancer (47 range from 35 to 56 years) at diagnosis. There was also no statistical difference in the median age between postmenopausal women with type 1 (62 range from 46 to 82 years) and type 2 endometrial cancer (62 range from 47 to 68 years) at diagnosis. The clinical characteristics of the patients with type 1 and type 2 endometrial cancer are summarised in Table 1.

### The serum levels of sex hormones were not different between women with type 1 and type 2 endometrial cancer

First we investigated the serum levels of E2, progesterone, testosterone, FSH and LH in patients. There were no statistically significant difference in the levels of E2, progesterone, testosterone, FSH and LH between women with type 1 and type 2 endometrial cancer (p > 0.05).

We then investigated the serum levels of E2, progesterone, testosterone, FSH and LH in patients taking into account cancer types and menopausal status (Table 2). There was no statistically significant difference in the levels of E2 (p = 0.611), progesterone (p = 0.931), testosterone (p = 0.988), FSH (p = 0.323) and LH (p = 0.304) between premenopausal women with type 1(n = 59) and type 2 (n = 8) endometrial cancer. In addition, there was also no statistically significant difference in the levels of E2 (p = 0.997), progesterone (p = 0.986), testosterone (p = 0.531), FSH (p = 0.218) and LH (p = 0.103) between postmenopausal women with type 1 (n = 104) and type 2 (n = 16) endometrial cancer.

We further investigated the number of patients with excess levels of E2, progesterone and testosterone, or lower levels of FSH and LH in postmenopausal patients with type 1 and type 2 endometrial cancer (Table 3). The number of patients with excess levels of E2, progesterone and testosterone, or lower levels of FSH and LH was 15%, 6%, 1%, 10% and 14%, respectively in postmenopausal women with type 1 endometrial cancer (n = 104). The number of patients with excess levels of E2, progesterone and testosterone, or lower levels of FSH and LH was 6%, 0%, 0%, 3% and 14%, respectively in postmenopausal women with type 2 endometrial cancer (n = 16). There was no statistically significant difference in the numbers of patients with abnormal range of these sex hormones between type 1 and type 2 endometrial cancer in postmenopausal women (p > 0.162).

We also investigated the numbers of premenopausal patients with abnormal ranges (lower or excess) of E2, progesterone, testosterone, FSH and LH in each type of endometrial cancer (Table 4). There was no statistically significant difference in the numbers of premenopausal patients with excess levels of E2, progesterone, testosterone or lower levels of FSH and LH between type 1 (n = 59) and type 2 (n = 8) endometrial cancer (p > 0.419).

### The levels of sex hormones were not associated with parity in endometrial cancer

A number of studies have reported that parity is negatively correlated with the incidence of endometrial cancer due to the changes of sex hormones^5^,^17^. We then investigated whether the levels of sex hormones were associated with parity in endometrial cancer. The levels of E2, progesterone, testosterone, FSH and LH were not different in premenopausal (n = 67) and postmenopausal patients (n = 127) with differing parity (Table 5, p > 0.05).

### The positivity of estrogen receptor (ER) and progesterone receptor (PR) in type 1 and type 2 endometrial cancer

A number of studies have suggested that the expression of ER or PR is associated with the survival rate and time of endometrial cancer^18^,^19^ and type 2 endometrial cancer is associated with a poorer prognosis due to the high grade^10^. We then investigated whether the expression of ER or PR was different between type 1 and type 2 endometrial cancer. In type 1 endometrial cancer (n = 157), 96% of cases were ER and PR positive, whereas in type 2 endometrial cancer (n = 22), 82% or 77% of cases were ER or PR positive respectively (Table 6). The positivity of ER or PR in type 1 endometrial cancer was significantly higher than that in type 2 endometrial cancer (p = 0.03 or 0.01, respectively, Table 6).

## Discussion

Unopposed estrogen exposure is associated with an increased risk of endometrial cancer^9^,^20^, in part because estrogen has a mitogenic effect on endometrial tissue, by stimulating the endometrial glands and stromal cells to grow and proliferate during the menstrual cycle^21^,^22^. Before the menopause, the ovaries are the major source of estrogen and progesterone production in response to LH and FSH secretion by the pituitary gland. In contrast, after the menopause, the ovaries stop synthesising estrogen and progesterone and as a result the extraglandular production of estrogen is unoppposed. In addition to ovarian production, estrogens are also produced through the peripheral aromatization of testosterone in adipose tissue, especially in obese women^23^.

Endometrial cancer is traditionally divided into estrogen dependent (type 1) and estrogen independent (type 2)^12^. However, the ages at diagnosis of type 1 and type 2 are different, and type 2 endometrial cancer usually occurs in older post-menopausal women who have reduced levels of estrogen, suggesting type 2 endometrial cancer may not be caused by unopposed excess estrogen^13^. This encouraged us to investigate whether the levels of sex hormones were different between the cancer types. Studies reported that the levels of sex hormones such as E2, testosterone, FSH and LH were altered in endometrial cancer, but the subtypes of endometrial cancer and menopausal status had not been taken into account^24^,^25^. Interestingly, in our current study, we found that there was no difference in circulating levels of E2, progesterone, testosterone, FSH and LH between patients with type 1 and type 2 endometrial cancer in either premenopausal or postmenopausal women.

Excess estrogen is one of the risk factors for developing type 1 endometrial cancer. In the EPIC study^25^, those authors found that sex hormones levels were not clearly associated with the risk of developing endometrial cancer in premenopausal women, but excess levels of E2 were associated with an increased endometrial cancer risk in postmenopausal women. However, in our study we found 20% and 13% of cases had excess estrogen in either premenopausal or postmenopausal patients respectively, suggesting that excess estrogen is not correlated with the menopausal status. In addition, we also found that the number of patients with excess estrogen was not different between type 1 and type 2 endometrial cancer regardless of menopausal status. Our data suggest that excess estrogen may be also involved in the pathogenesis of type 2 endometrial cancer. The difference between the EPIC study and our study could be due to the ethnicity of the study population. We have recently reported that in comparison to Caucasian populations, Chinese populations have a higher incidence of both type 1 and type 2 endometrial cancer prior to the menopause^26^.

Endocrine signallings between the hypothalamus, anterior pituitary gland and ovaries regulate the female reproductive system. It is well known that FSH and LH promote ovulation and stimulate secretion of estrogen and progesterone from the ovaries before the menopause. A number of studies have also suggested that FSH, LH and testosterone are involved in the pathogenesis of endometrial cancer via their ability to regulate endometrial cancer cell growth^24^,^25^,^27^. In this study, our data show that the numbers of premenopausal patients with excess levels of FSH or LH were higher in both type 1 and type 2 endometrial cancer, suggesting that excess FSH or LH contributes to higher levels of estrogen in endometrial cancer. However, the numbers of premenopausal women with excess FSH or LH were not different between type 1 and type 2 endometrial cancer.

It is well documented that parity is negatively correlated with the incidence of endometrial cancer^5^,^17^. This has been thought to result from a shift in hormonal balance of estrogen and progesterone that resulting in a reduction of unopposed estrogen and lack of ovulation during pregnancy^5^,^8^. However this may not be the case^17^,^28^. In our study, our data show that the levels of sex hormones including E2, progesterone, testosterone, FSH and LH were not correlated with parity regardless of menopausal status, suggesting that parity may not be associated with the changes in sex hormone levels in endometrial cancer. This suggests that the protective effect of pregnancy against endometrial cancer maybe mediated by non-endocrine mechanisms.

Estrogen receptor (ER) and/or progesterone receptor (PR) positivity is associated with better prognosis (or survival) of endometrial cancer^18^,^19^. Because type 2 endometrial cancer has a poorer prognosis and lower survival rate than type 1 cancer, we then questioned whether there was a difference in ER or PR positivity between the types of endometrial cancer. Interestingly in this study we found although the rate of positivity of ER (96%) or PR (96%) in type1 was significantly higher than that in type 2 endometrial cancer, but the majority of type 2 endometrial cancer were still ER (82%) or PR (77%) positive. In addition, the upper confidence limits of ER or PR positivity in both type 1 and type 2 are very close (98.5%, or 94.8% respectively, Table 6). This result suggests that the positivity of ER or PR may not be related to the subtypes of endometrial cancer. Due to the sample size, we were not able to subdivide our study population into premenopausal and postmenopausal women to investigate ER or PR positivity in type 1 and type 2 endometrial cancers.

The incidence of type 1 endometrial cancer is approximately 80% to 85%^12^, while type 2 endometrial cancer represents 10–15% worldwide^12^. In our current study the distribution of type 1 and 2 endometrial cancer is similar to the incidence of subtypes of endometrial cancer reported in the literature. However, despite of the collection of samples over 5 years in one women’s hospital, the number of type 2 endometrial cancer was still small. To increase the power, the conclusions drawn from this study would need to be further studied with large sample size.

There are some limitations in this study. First, the age of menopause was self-reported. Secondly, the levels of sex hormones and characteristics of the women with endometrial cancer and its histological subtypes maybe dependent on ethnicity^29^,^30^, and this study comprised patients only of Han Chinese ethnicity.

In conclusion, to our knowledge, this is the first study to investigate sex hormones levels in women with endometrial cancer, taking into account cancer types and menopausal status. We demonstrate that there is no difference in the levels of estrogen, progesterone, testosterone, FSH or LH between type 1 and type 2 endometrial cancers regardless of menopausal status. We also find that parity is not associated with the levels of sex hormones in endometrial cancer regardless of menopausal status. We further find that although the frequency of ER or PR positivity in type 1 endometrial cancer is higher than that in type 2 endometrial cancer, the majority of type 2 endometrial cancer were also ER or PR positive. Taken together, this suggests that type 2 endometrial cancer is not completely estrogen independent, and type 1 and type 2 endometrial cancer may have similar pathogenesis.

## Materials and Methods

This study was approved by the Ethics Committee of Wuxi Maternity and Children Hospital, Nanjing Medical University, Wuxi, China. All patient-derived blood samples and tissues were obtained with written informed consent.

All methods were performed in accordance with the relevant guidelines and regulations.

### Study participants

There were in total 389 patients with a primary diagnosis of endometrial cancer from 2010 to 2015 at Wuxi Maternity and Children Hospital, Nanjing Medical University, which serves a diverse urban and rural population in China. In this study, only 187 women with a primary diagnosis of endometrial cancer consented to donate blood and tissue samples. All clinical data were collected from the electronic based medical records of patients and clinical characteristics included age at diagnosis, self-reported age at menopause, parity and pathological findings. No patients had taken any estrogen replacement therapy. Of them, 163 patients were diagnosed with type 1 endometrial cancer and 24 patients were diagnosed with type 2 endometrial cancer.

Endometrial cancer was diagnosed first by a physical examination and then endometrial biopsy. The endometrial tissue was examined histologically for characteristics of cancer including types of cancer.

The classification of type 1 or type 2 endometrial cancer was determined by pathological examination of hysterectomy specimen, including cancer histologic subtypes and grades. We classified endometrioid and adenosquamous carcinoma with grade 1 and 2 as type 1 endometrial cancer. Clear-cell, serous, mucinous carcinoma and grade 3 endometrioid carcinomata were classified as type 2 endometrial cancers, according to the classification of the International Federation of Gynaecology and Obstetrics (FIGO).

### Collection of blood sample for hormone analysis

Blood samples from 187 women who were diagnosed with primary endometrial cancer (type 1, n = 163, type 2, n = 24) were collected by venepuncture into plain Vaccutainer^®^ tubes prior to any treatment. For premenopausal women, blood samples were collected between 3 and 5 days after the end of menstruation (follicular phase). All the blood samples were allowed to clot, centrifuged at 2500 × *g* and the serum was aspirated and stored in aliquots at −80 °C.

### Determination of the levels of estradiol (E2), progesterone, testosterone, follicle stimulating hormone (FSH) and luteinizing hormone (LH)

The serum levels of E2, progesterone, testosterone, FSH and LH in women with endometrial cancer were measured using ELISA kit following the manufacturer’s instructions (Beckman Coulter, USA). According to each ELISA kit, the reference range for E2, progesterone, FSH, LH and testosterone in follicular phase are 27–123 pg/ml, 0.31–1.52 ng/ml, 3.85–8.78 IU/L, 2.12–10.89 IU/L and 0.06–0.82 ng/ml, respectively. The reference range for E2, progesterone, FSH, LH and testosterone in postmenopausal women are <37 pg/ml, 78 ng/ml, >20 IU/L, >10 IU/L and <0.75 ng/ml, respectively.

### Immunohistochemistry

The expression of estrogen receptors (ER) and progesterone receptor (PR) in endometrial tissue (n = 179) was measured by immunohistochemistry on paraffin-embedded sections. Briefly, antigen retrieval was performed by treatment with citric acid (pH 6.0) for 15 min. Non-specific antibody binding was blocked by incubating with 10% fetal calf serum for 20 min. Rabbit anti-human ER or PR monoclonal antibody (1:100, Jingqiao Corp, Beijing China) were added for 1 h at room temperature. Sections were then washed with phosphate-buffered saline and incubated with HRP-Polymer anti-Rabbit IgG (Maxvision 2 kit, Maxim BIO, Fuzhou, China) for 15 minutes. The antigen–antibody complexes were visualised using 3,3-Diaminobenzidine and counterstained with haematoxylin. The cut-off point of 1% positive cells was considered as ER or PR positive.

### Statistical analysis

The statistical difference in E2, progesterone, testosterone, FSH and LH in the number of patients with type 1 or type 2 endometrial cancer in premenopausal and postmenopausal women was assessed by Fisher’s Exact test using the Prism software package. A Mann-Whitney U-test was used to assess the statistical differences in the serum levels of individual hormones between type 1 and type 2 endometrial cancer by Prism software with *p* < 0.05 being considered as statistically significant.

## Additional Information

**How to cite this article:** Wan, J. *et al*. The levels of the sex hormones are not different between type 1 and type 2 endometrial cancer. *Sci. Rep.* **6**, 39744; doi: 10.1038/srep39744 (2016).

**Publisher's note:** Springer Nature remains neutral with regard to jurisdictional claims in published maps and institutional affiliations.

## Figures and Tables

**Table 1:** Clinical characteristics of the study population.

	Women with type 1 endometrial cancer (N = 163)	Women with type 2 endometrial cancer (N = 24)
Age at diagnosis (years, median/range)	55(24–82)	56 (35–74)
Premenopause (number, %)	59 (36.2%)	8 (33.3%)
Post- menopause (number, %)	104 (63.8%)	16 (66.7%)
Parity (number, %)		
0	7 (4.3%)	1 (4%)
1	90 (55.2%)	15 (63%)
2	53 (32.5%)	7 (29%)
≥3	13 (8%)	1 (4%)
BMI (mean ± SD, kg/m^2^)	24.32 ± 3.84	25.45 ± 3.55

**Table 2:** The levels of sex hormones in premenopausal and postmenopausal women with type1 or type 2 endometrial cancer.

	E2 (pg/ml) (median/range)	Progesterone (ng/ml) (median/range)	Testosterone (ng/ml) (median/range)	FSH (IU/L) (median/range)	LH (IU/L) (median/range)
**Premenopause**					
Type 1 (n = 59)	52.8 (5–323)	0.46 (0.08–31.5)	0.22 (0.02–1.05)	8.37 (1.34–86.2)	7.16 (0.62–40.8)
Type 2 (n = 8)	27 (9.6–166)	0.50 (0.38–0.58)	0.24 (0.1–0.29)	16.4 (4–86.1)	8.7 (3–50.0)
P value	P = 0.611	P = 0.931	P = 0.988	P = 0.323	P = 0.304
**Postmenopause**					
Type 1(n = 104)	15.7 (5–221)	0.34 (0.03–8.64)	0.20 (0.02–0.93)	47.2 (3.4–93.5)	22.8 (4.1–54.9)
Type 2 (n = 16)	15.6 (5–48.3)	0.35 (0.16–0.71)	0.21 (0.05–0.33)	38.8 (6.6–95.1)	17.0 (4.3–38.6)
P value	P = 0.992	P = 0.986	P = 0.531	P = 0.228	P = 0.103

**Table 3:** The number of postmenopausal patients with excess levels of E2, progesterone and testosterone or lower levels of FSH and LH.

Postmenopause	Excess E2	Excess Progesterone	Excess Testosterone	Lower FSH	Lower LH
Type 1(n = 104) (number, %)	15 (14%)	6 (5.3%)	1(1%)	10 (9.5%)	14 (13.5%)
Type 2 (n = 16) (number, %)	1 (6.2%)	0 (0%)	0 (0%)	3 (18.7%)	2 (12.5%)
P value	P = 0.634	P = 0.511	P = 0.896	P = 0.162	P = 0.733

**Table 4:** The number of premenopausal women with excess of E2, progesterone, testosterone, FSH and LH in type 1 and type 2 endometrial cancers.

	Excess reference range (number, %)	P value
**E2**		
Type 1 (n = 59)	12 (20.3%)	0.672
Type 2 (n = 8)	1 (12.5%)	
**Progesterone**		
Type 1 (n = 59)	4 (6.8%)	0.884
Type 2 (n = 8)	0 (0%)	
**Testosterone**		
Type 1 (n = 59)	1 (1.7%)	0.419
Type 2 (n = 8)	0 (0%)	
**FSH**		
Type 1 (n = 59)	29 (49.1%)	0.511
Type 2 (n = 8)	5 (62.5%)	
**LH**		
Type 1 (n = 59)	21 (35.6%)	0.903
Type 2 (n = 8)	3 (37.5%)	

**Table 5:** The association between parity and the levels of sex hormones in premenopausal and postmenopausal patients with endometrial cancers.

	E2 (pg/ml) (median/range)	Progesterone (ng/ml) (median/range)	Testosterone (ng/ml) (median/range)	FSH (IU/L) (median/range)	LH (IU/L) (median/range)
**Premenopause**					
Parity					
0 (n = 8)	24.61 (5–178.1)	0.3375 (0.08–0.693)	0.177 (0.025–0.547)	18.26 (5.1–33.84)	15.96 (2.56–34.67)
1 (n = 51)	61.42 (5–323.5)	0.478 (0.158–31.51)	0.224 (0.025–1.05)	7.05 (1.39–86.24)	7.04 (0.62–50.04)
2 (n = 8)	73.65 (9.6–137.7)	0.671 (0.134–0.921)	0.225 (0.11–0.328)	5.85 (1.34–57.36)	6.83 (3.97–35.32)
P value	>0.05	>0.05	>0.05	>0.05	>0.05
**Postmenopause**					
Parity					
1 (n = 54)	13.95 (5–98.9)	0.362 (0.05–0.865)	0.211 (0.025–0.935)	43.75 (7.32–95.01)	21.88 (4.13–48.51)
2 (n = 53)	19.47 (5–221.9)	0.348 (0.03–8.64)	0.226 (0.025–0.551)	46.17 (3.42–93.53)	22.5 (5.28–54.96)
≥3 (n = 13)	13.7 (5–48.33)	0.201 (0.03–0.477)	0.129 (0.025–0.309)	58.77 (17.42–90.38)	25.95 (6.64–51.93)
P value	>0.05	>0.05	>0.05	>0.05	>0.05

**Table 6:** The positivity of estrogen receptor (ER) and progesterone receptor (PR) in endometrial cancers.

	ER positive (number, %, lower, upper CL)	PR positive (number, %, lower, upper CL)
Type 1 (n = 157)	151 (96%) (91.8%, 98.5%)	151 (96%) (91.8%, 98.5%)
Type 2 (n = 22)	18 (82%) (59.7%, 94.8%)	17 (77%) (54.6%, 92.2%)
P value	0.03	0.01

^*^data missing in 6 cases with type 1endometrial cancer, and 2 cases with type 2 endometrial cancer.
